# Prevalence of suicidality and associated factors of suicide risk in a representative community sample of families in three East African refugee camps

**DOI:** 10.1007/s00127-023-02506-z

**Published:** 2023-06-05

**Authors:** Florian Scharpf, Faustine Bwire Masath, Getrude Mkinga, Edna Kyaruzi, Mabula Nkuba, Maregesi Machumu, Tobias Hecker

**Affiliations:** 1 Department of Psychology, Bielefeld University, P. O. Box 100131, 33501 Bielefeld, Germany; 2 Institute for Interdisciplinary Research on Conflict and Violence, Bielefeld University, P. O. Box 100131, 33501 Bielefeld, Germany; 3 Department of Educational Psychology and Curriculum Studies, Dar es Salaam University College of Education, P. O. Box 2329, Dar es Salaam, Tanzania

**Keywords:** Suicidal ideation, Suicidal behaviour, Refugees, Trauma, Social support

## Abstract

**Purpose:**

To assess the prevalence of suicidality and associated factors of suicide risk in a sample of Burundian refugee families living in three refugee camps in Tanzania.

**Methods:**

Children (*n* = 230) and their parents (*n* = 460) were randomly selected and interviewed about suicidality (suicidal ideation, plans, and attempts) and a range of sociodemographic, psychological, and environmental factors. Multinomial logistic regression analyses were conducted to examine factors associated with children and parents’ lower and moderate or high current suicide risk.

**Results:**

Past-month prevalence of suicidal ideation, plans, and attempts were 11.3%, 0.9% and 0.9%, respectively, among children; 37.4%, 7.4% and 5.2%, respectively, among mothers; and 29.6%, 4.8% and 1.7%, respectively, among fathers. Older age in years (aOR_lower_ = 2.20, 95% CI 1.38–3.51; aOR_moderate/high_ = 3.03, 95% CI 1.15–7.99) and higher levels of posttraumatic stress disorder symptoms (aOR_lower_ = 1.64, 95% CI 1.05–2.57; aOR_moderate/high_ = 2.30, 95% CI: 1.02–5.16), internalizing (aOR_moderate/high_ = 2.88, 95% CI 1.33–6.26) and externalizing problems (aOR_lower_ = 1.56, 95% CI: 1.06–2.31; aOR_moderate/high_ = 3.03, 95% CI 1.42–6.49) were significantly positively associated with children’s current suicide risk. For mothers, higher perceived instrumental social support (aOR_moderate/high_ = 0.05, 95% CI < 0.01–0.58) was significantly negatively related to suicide risk, whereas exposure to community violence (aOR_lower_ = 1.97, 95% CI 1.30–2.99; aOR_moderate/high_ = 1.59, 95% CI 1.00–2.52), living in larger households (aOR_lower_ = 1.74, 95% CI 1.17–2.57), and higher psychological distress (aOR_moderate/high_ = 1.67, 95% CI 1.05–2.67) were significantly positively associated with suicide risk. For fathers, higher perceived instrumental social support (aOR_moderate/high_ = 0.04, 95% CI < 0.01–0.44) and having more years of formal education (aOR_moderate/high_ = 0.58, 95% CI 0.34–0.98) were significantly negatively and exposure to war-related trauma (aOR_moderate/high_ = 1.81, 95% CI 1.03–3.19) was significantly positively associated with suicide risk.

**Conclusion:**

Prevention programs should target psychopathology, community violence and social support to mitigate children and parents’ current suicide risk.

**Supplementary Information:**

The online version contains supplementary material available at 10.1007/s00127-023-02506-z.

## Introduction

Suicide is a serious public health problem, with over 700,000 people dying by suicide each year [1]. Existing research has identified a range of risk factors for adults’ suicidal ideation and behaviour including being female, younger age, a lower educational level and socioeconomic status as well as current psychopathology [2, 3]. Among youth, experiencing maltreatment by parents and bullying by peers appear to be particularly salient risk factors [4, 5]. Suicidal behaviour has significant long-term negative effects for the health and psychosocial well-being of affected individuals [6, 7] as well as of their families and friends [8].

Although about 80% of suicides occur in low- and middle-income countries [9], only 10% of all published research on suicidality is conducted in these settings [10]. However, evidence suggests that the nature and course of suicidality among people in low- and middle-income countries may be different from those in high-income countries. Notably, whereas studies in high-income countries typically find higher prevalence rates of suicidal ideations compared to actual suicidal behavior including plans and attempts [11, 12], differences between the prevalence rates of suicidal ideation, suicide plans and attempts are less pronounced in low- and middle-income countries [3, 13, 14]. While it is possible that stigma and taboos around suicidality decrease individuals` willingness to disclose suicidal thoughts before they engage in suicidal behaviour, such findings could also point to altered patterns in the transition from suicidal ideation to suicidal behaviours among populations in lower-income settings [15]. Moreover, the nature of and extent to which specific factors explain suicidality in high-income countries and low-and middle-income countries may also differ. For instance, meta-analytic evidence suggests a lower association between psychopathology and suicidality in low-and middle-income countries compared to high-income countries [16], which highlights the importance of understanding how contextual and interpersonal factors, such as economic hardship, social support, family and community violence, contribute to individuals` suicide risk in resource-constrained settings. Although there is considerable economic, sociocultural and political heterogeneity among low- and middle-income countries, there is a great need for more research on the prevalence and correlates of suicidality among representative at-risk samples in these settings to improve the generalizability and effectiveness of suicide prevention efforts [10, 17].

Refugees may be at a particularly high risk of suicidality because established risk factors such as mental illness, family conflicts, social isolation and economic strain are exacerbated in this population [18, 19]. Moreover, many refugees have been directly or indirectly exposed to war-related traumatic events, which has been associated with increased suicidality [20, 21]. Refugees living in camps in low-resource regions may be particularly vulnerable because they face unique challenges related to their living environment, such as high levels of family and community violence, disruption of social support networks, overcrowded housing, lack of basic necessities as well as exclusion from work and educational opportunities [22–24]. Most existing studies examining the prevalence and associated factors of suicidality among refugees have been conducted in high-income countries, although about 85% of all refugees live in low- and middle-income countries mainly in Sub-Saharan Africa, the Middle East, and South Asia [25], and a considerable number of them in refugee camps [26].

Recent meta-analyses and systematic reviews summarize the current state of research on the prevalence of suicidality among refugee populations [27–30]. A meta-analysis of 11 studies with community samples of refugees aged 16 or older reported periodic prevalence rates of 20.5% for suicidal ideation and of 0.57% for suicide attempts [27]. Cogo and colleagues [28] included 77 studies (thereof 54 with community samples) with five different subgroups of displaced individuals of all age groups: refugees granted permanent asylum, refugees living in camps or with only temporary protection, asylum seekers, mixed samples of refugees and asylum seekers, and internally displaced people. In community samples of all subgroups, the prevalence of suicidal ideation and suicide attempts ranged from 0.17 to 70.6% and from 0.14 to 15.1% for suicide attempts, respectively. Among refugees living in camps, the prevalence of suicidal ideation and suicide attempts ranged from 1.2 to 32.3% and from 0.18 to 7.3%, respectively [28]. A systematic review of the evidence on the prevalence of mental health problems among refugee youth in European countries reported a point prevalence of 5% for suicidal ideation and behaviour across four studies [29]. Among refugee youth living in camps, the prevalence of suicidal ideation ranged from 4.4 up to 70%, with four out of five studies reporting rates of at least 20% [28, 30]. However, findings on whether refugees are actually at higher risk of suicidality than host populations have been equivocal and seem to depend on the specific context. Whereas four out of five studies in the review by Cogo et al. suggest a lower suicide risk for refugees granted asylum in high-income countries compared to host populations, two out of three studies point to a higher risk of suicidal ideation among refugees living in camps in low- and middle-income countries compared to host populations [28].

While the interpretability and comparability of the varying prevalence rates on suicidality strongly depend on contextual, e.g., the study setting and background of the sample, and methodological factors, e.g., the timeframe and formulation of the questions, a prominent gap in the current evidence on suicidality among refugees is the striking lack of studies focusing on refugees living in camps, which is also emphasized by Cogo et al. in their systematic review [28]. Among the overall ten studies with refugees living in camps in the meta-analysis by Haase et al. [27] and the systematic review by Cogo et al. [28], only three studies were conducted in refugee camps in Sub-Saharan Africa. Furthermore, within refugee camps there is particularly scarce evidence for men, with only five out of nine studies with adults including men, as well as for children and adolescents. Finally, there is a dearth of research focusing on families and examining possible differential associations between contributing factors and suicide risk among parents and children.

In an effort to contribute to closing these gaps, the present study examined the prevalence of suicidality among Burundian children and their parents living in refugee camps in Western Tanzania. While establishing prevalence rates of suicidal ideation and behaviour is crucial to map the scope of the problem in this vulnerable and understudied population, the identification and modification of locally relevant risk and protective factors are substantial for developing and implementing prevention efforts. Therefore, this study also examined which factors were associated with the current suicide risk of children and parents. The inclusion of families allowed us to shed light on potential differential associations between contributing factors and suicide risk for children, mothers, and fathers. We anticipated a high prevalence of suicidality among family members. We also hypothesized that past and current experiences of violence as well as higher levels of psychopathology would be related to an increased suicide risk, whereas higher social support would be associated with reduced suicide risk.

## Methods

### Study setting

This study was part of a larger cross-sectional study which examined the prevalence and correlates of traumatic experiences and mental health problems of Burundian refugees in Tanzania. Data collection took place between January and May 2018 in three refugee camps in Western Tanzania. In 2015, Burundi was plunged into a political conflict when civil protests were put down with severe violence and human rights violations. More than 400,000 Burundians fled to neighboring countries including Tanzania.

### Sampling and participants

A systematic random sampling approach was applied. In each of the three camps, two zones were randomly selected. Next a sampling direction was randomly determined by spinning a pen at the center of the selected zones. Every sixth tent or hut in this direction was approached. Families were eligible to participate if they consisted of a father or primary male caregiver, a mother or primary female caregiver and a child in primary school age, i.e., between 7 and 15 years. This inclusion criterion allowed for a variety of possible constellations within families, considering biological parents as well as other adults, e.g., relatives or foster parents, as caregivers. For the sake of clarity, caregivers are referred to as parents, mothers or fathers in this article. If there were more eligible children in a family, the oldest child in that age range was considered. This approach resulted in a sample of 230 family triads with overall 690 participants (460 parents, 230 children).

### Procedures

The selected families were invited to come to the compound of a collaborating non-governmental organization within the camp. The parents provided informed written consent for their own participation and that of their child. Children aged 11 and older were also asked for their written informed consent, while oral assent was sought from younger children. All but two of the invited families agreed to participate. Separate structured clinical interviews were conducted with each parent and the child in a discrete setting. The interviewers were Tanzanian master-level psychologists and research assistants from the refugee community in the camp. The research assistants had received one week of training on handling the study instruments and important principles of conducting interviews. As Kiswahili is the lingua franca in Tanzania and in the refugee camps, all measures used in the structured clinical interviews had been translated from English to Kiswahili using blind back-translation or existing Kiswahili versions were used. The interviews were conducted either in Kiswahili or in Kirundi, the native language of Burundians, if participants did not feel comfortable expressing themselves in Kiswahili. If the latter was the case, the interviewers from the refugee community translated the questions in an ad hoc manner, while the Tanzanian interviewers were supported by trained interpreters from the refugee community. To ensure that interviewers and interpreters used the same expressions and terms in their translations, their training focused on the adequate translations of mental health terms and concepts in Kirundi. After completing the interviews, families received approximately 8 US-Dollars as a compensation for their time and efforts. For participants whose interviews revealed an increased suicide risk, a referral system was established to provide these participants with timely psychological support. More details on the extensive study procedures can be found elsewhere [31, 32].

### Measures

More detailed descriptions of the study measures can be found in Supplementary File 1.

#### Suicidality

Parents and children’s suicidality was assessed using the Suicidal Scale of the Mini-International Neuropsychiatric Interview [MINI; 33] and the MINI for Children and Adolescents [MINI-KID; 34], respectively. The eight items of the MINI are answered with “No” or “Yes” and they are weighted according to their estimated contribution to the suicide risk. Seven items refer to the past month and assess passive and active forms of suicidal ideation (3 items; Yes = 1, 2 and 6 points), suicide plans (Yes = 8 points), active preparations for self-injury or a suicide attempt (Yes = 9 points), non-suicidal self-injury (Yes = 4 points) and suicide attempt (Yes = 10 points). One item assesses whether a person attempted suicide in their lifetime (Yes = 4 points). If at least one of the items about suicidal ideation is answered “Yes”, the frequency (occasionally, often, very often) and intensity (mild, moderate, severe) of the ideation are assessed and the respondent indicates whether they can control these thoughts or not (No = 8 points). If all items on suicidal ideation in the past month are answered “No”, the remaining questions referring to the past month are skipped, and the respondent is directly asked about suicide attempts in their lifetime. The points of all items are summed up to determine the respondent’s current suicide risk: low (1 to 8 points), moderate (9 to 16 points) or high (≥ 17 points). In the MINI-KID, the suicide scale consists of six items. Three items assess lifetime suicidal ideation (Yes = 1 point), self-injury (Yes = 2 points) and suicide attempt (Yes = 4 points). If all these items are answered “No”, the five following items referring to the past month are skipped. These items assess whether the child had a death wish (Yes = 1 point), wanted to hurt himself/herself (Yes = 2 points), thought about killing himself/herself (Yes = 6 points), had suicide plans (Yes = 10 points) and attempted suicide (Yes = 10 points) in the past month. The current suicide risk is defined as low (1 to 5 points), moderate (6 to 9 points) or high (≥ 10 points).

#### Sociodemographic characteristics

We assessed parents` age, level of education, household size and average income per month in Tanzanian Shillings (TZS; 1 US Dollar = approx. 2400 TZS). The latter variable was collapsed into the three categories: 0 to 5000 TZS (0), 6000 to 20,000 TZS (1) and more than 20,000 TZS (2). Children answered purpose-built questions about their age, their current school attendance, and their orphan status (1 = No orphan/2 = Half- or full orphan).

#### Social support

Parents’ instrumental social support was assessed through two purpose-built questions asking how much people they could turn to for help if they (a) suddenly needed a small amount of money and (b) they faced a long-term emergency such as harvest failure: no one (0), one or two people (1), three or four people (2), five or more people (3). The responses to both questions were summed up. Children’s social support was operationalized through the purpose-built question “*How many good friends do you have?”.*

#### Exposure to war-related traumatic events

A checklist adapted from Ertl et al. [35] was used to assess children and parents’ lifetime exposure to various events they may have directly experienced, witnessed or perpetrated themselves in the context of war. The checklist for children contained 22 events and the checklist for parents consisted of 31 events.

#### Current exposure to violence

Children’s current exposure to violence was operationalized as children’s self-reported experiences of maltreatment by their parents and assessed using the Parent–Child Conflict Tactic Scales [CTSPC; 36]. For the analysis, a sum score of child maltreatment by mother and father was used (Cronbach’s alpha = 0.91).

Parents’ reports of their directly experienced and witnessed incidents of community violence, e.g., physical, and sexual assault, were used to operationalize parents’ current exposure to violence. The nine items of the respective checklist were summed up for the analysis.

#### PTSD symptoms

Children’ PTSD symptoms according to the fifth edition of the Diagnostic and Statistical Manual of Mental Disorders (DSM-5) were assessed using the University of California at Los Angeles Child/Adolescent PTSD Reaction Index UCLA RI-5 [37]. The total score of PTSD symptoms used for analyses had a high internal consistency (Cronbach`s alpha = 0.90).

The PTSD Checklist for DSM-5 [PCL-5; 38] was used to assess mothers and fathers` PTSD symptoms. The total score of PTSD symptoms had a high internal consistency both for mothers (Cronbach`s alpha = 0.94) and fathers (Cronbach`s alpha = 0.91).

#### Other mental health problems

Children`s internalizing and externalizing problems were assessed using the self-report version of the Strengths and Difficulties Questionnaire [SDQ; 39]. The subscales emotional problems and peer problems were combined into an internalizing problems score (Cronbach`s Alpha = 0.57), while the subscales conduct problems and hyperactivity formed an externalizing problems score (Cronbach`s alpha = 0.54). 

The 18-item version of the Brief Symptom Inventory [BSI-18; 40] served as a measure of parents` psychological distress, assessing symptoms of depression, anxiety and somatization with six items each. The sum score showed high internal consistency both for mothers (Cronbach`s alpha = 0.92) and fathers (Cronbach`s alpha = 0.90).

In addition, we assessed parents’ substance use with the Alcohol, Smoking and Substance Involvement Screening Test (ASSIST) [41]. We considered the substances tobacco products, alcoholic beverages, cannabis, and other substances the participants could specify (e.g., Khat). The total substance involvement score had high internal consistency for mothers (Cronbach`s Alpha = 0.87) and fathers (Cronbach`s Alpha = 0.90).

### Statistical analyses

We descriptively examined mothers, fathers, and children’s responses to the suicide scale of the MINI and MINI-KID to determine the prevalence of suicidality. For the following analyses, we retained outliers defined as values deviating more than three standard deviations from the mean in the clinical variables related to exposure to violence and mental health problems to consider the whole range of naturally occurring experiences. However, we removed such extreme values in the non-clinical variable social support, specifically one child, three mothers and four fathers. We computed bivariate correlations between the sociodemographic, psychological, and environmental variables used to predict participants’ current suicide risk. Next, we conducted multinomial logistic regression analyses to examine associations between independent variables and the dependent variable current suicide risk. One child was excluded from these analyses because she had lived with her foster parents for only a short period of time and so no reliable information about experiences of maltreatment could be obtained. To be able to detect possible multivariate effects, we included all independent variables in the regression analyses rather than selecting them based on univariate analyses. Separate regression models were calculated for mothers, fathers, and children. The outcome variable current suicide risk had a maximum of four levels: no risk, which served as a reference group, low risk, moderate risk and high risk. However, given relatively low numbers of children, mothers and fathers with a moderate and high risk, these levels were merged to a *moderate/high risk* level. All independent variables were standardized for the logistic regression analyses to ease interpretation. Results of the logistic regression analyses were reported as adjusted odds ratios with 95% confidence intervals. Nagelkerke *R*^2^ (range 0–1) was used to indicate the degree of improvement in fit of each fitted model compared to the baseline model (0–0.1: poor improvement in fit, 0.1–0.3: modest improvement, 0.3–05: moderate improvement, more than 0.5: strong improvement) [42]. A two-tailed *α* = 0.05 was used to evaluate the significance of associations. All analyses were conducted with SPSS version 27.

## Results

### Descriptive statistics

Table 1 shows important sociodemographic characteristics of the participating families. The descriptive statistics of study variables are displayed in Table 2, while bivariate correlations between study variables for the models of children, mothers and fathers can be found in Supplementary Tables 1–3.

**Table 1:** Sociodemographic characteristics of participating families

	Families (*N* = 230)			
	Children (*N* = 230)		Caregivers (*N* = 460)	
	Girls (*n* = 109)	Boys (*n* = 121)	Female (*n* = 230)	Male (*n* = 230)
Age, M (SD)	12.11 (2.03)		34.49 (8.48)	41.52 (11.00)
Age range	7–15		19–80	
Level of education, % (*n*)^a^				
No schooling	9.6 (22)		34.8 (80)	23.0 (53)
Primary school				
Class 1	11.7 (27)		2.2 (5)	3.5 (8)
Class 2	20.9 (48)		10.4 (24)	4.8 (11)
Class 3	16.5 (38)		10.0 (23)	8.3 (19)
Class 4	16.1 (37)		7.0 (16)	6.1 (14)
Class 5	14.3 (33)		11.7 (27)	14.8 (34)
Class 6	7.8 (18)		11.3 (26)	18.3 (42)
Some secondary school	3.0 (7)		11.7 (27)	16.9 (39)
Completed secondary school			0.9 (2)	4.3 (10)
Relationship to the child, % (*n*)				
Biological Parent			84.3 (194)	83.0 (191)
Aunt/uncle			1.3 (3)	1.7 (4)
Sister/brother			2.2 (5)	2.2 (5)
Other relative			2.5 (6)	1.7 (4)
Stepparent			3.0 (7)	3.9 (9)
Foster parent			6.5 (15)	7.4 (17)
Number of own children, M (SD)			5.21 (2.18)	5.35 (2.46)
Orphan (half or full orphan), % (*n*)	14.3 (33)			
Number of people in household, % (*n*)^b^				
3 to 5			15.7 (36)	
6 or 7			45.2 (104)	
8 or 9			24.8 (57)	
10 or more			14.3 (33)	
Average household income *p* month (TZS)^c^				
No income			32.2 (74)	
Up to 5000			25.7 (59)	
Up to 20,000			23.0 (53)	
More than 20,000			19.1 (44)	

* TZS Tanzanian Shilling*

^a^Children`s responses referred to their current level, caregivers` responses to the highest level they achieved. ^b^Information on household variables is averaged across mothers` and fathers` reports. ^c^1000TZS = ca. 0.4$

**Table 2:** Descriptive statistics of non-sociodemographic predictor variables for logistic regression models

	M	SD	Range
Child variables (*N* = 230)			
Number of close friends	4.04	2.52	0–18
War-related traumatic events	3.67	3.68	0–16
Child maltreatment (CTSPC)	82.72	93.53	0–784
PTSD symptoms (UCLA RI-5)	14.59	11.33	0–49
Internalizing problems (SDQ)	6.33	3.26	0–16
Externalizing problems (SDQ)	4.32	2.92	0–15
Mother variables (*N* = 230)			
Number of people to ask for material support	2.43	4.37	0–40
War-related traumatic events	13.50	5.36	0–23
Exposure to community violence	2.62	2.48	0–9
PTSD symptoms (PCL-5)	38.51	19.31	0–80
Psychological Distress (BSI-18)	30.12	15.68	0–67
Substance use (ASSIST)	6.83	10.91	0–80
Father variables (*N* = 230)			
Number of people to ask for material support	5.14	12.30	0–120
War-related traumatic events	16.80	4.88	1–29
Exposure to community violence	3.56	2.51	0–9
PTSD symptoms (PCL-5)	33.04	16.51	0–74
Psychological Distress (BSI-18)	23.48	13.74	0–63
Substance use (ASSIST)	15.80	15.97	0–72

*CTSPC*  conflict tactic scale parent–child, *UCLA RI-5*  University of California at Los Angeles Posttraumatic Stress Disorder Reaction Index for *DSM-5,* *SDQ* strengths and difficulties questionnaire, *PCL-5 PTSD* checklist for DSM-5, *BSI-18* brief symptoms inventory, *ASSIST*   alcohol, smoking and substance involvement screening test

### Prevalence of suicidality

Children and parents’ responses to the items of the MINI and MINI-KID suicide scales are shown in Table 3. Overall, the prevalence of suicidality (≥ 1 point) among children was 25.2% (*n* = 58). Most of those children fell in the lower risk category (*n* = 44), while nine children had a moderate and five children a high current suicide risk. Twenty-six children (11.3%) reported suicidal thoughts in the past month, while two children (0.9%) reported having a suicide plan and attempting suicide in the past month. The lifetime prevalence rates of suicidal ideation and suicide attempt were 24.7% (*n* = 57) and 4.3% (*n* = 10), respectively.

**Table 3:** Prevalence of suicidality among children, mothers and fathers

	Children (*N* = 230)		Mothers (*N* = 230)		Fathers (*N* = 230)	
	*n*	%	*n*	%	*n*	%
Past month						
Did you wish you were dead?	18	7.8	86	37.4	67	29.1
Did you want to hurt yourself?	7	3.0	27	11.7	19	8.3
Did you think about killing yourself?	10	4.3	47	20.4	27	11.7
Did you have a plan how to kill yourself?	2	0.9	17	7.4	11	4.8
Did you take any active preparations to injure or kill yourself?			14	6.1	8	3.5
Did you deliberately injure yourself without intending to kill yourself?			6	2.6	6	2.6
Did you try to kill yourself?	2	0.9	12	5.2	4	1.7
Lifetime:						
Did you wish you were dead?	54	23.5				
Did you want to hurt yourself?	15	6.5				
Did you try to kill yourself?	9	3.9	50	21.7	27	11.7
Current suicide risk (points in MINI)						
No risk (0 points)	172	74.8	124	53.9	145	63.0
Low risk (children: 1–5, parents: 1–8)	44	19.1	62	27.0	53	23.0
Moderate risk (children: 6–9, parents: 9–16)	9	3.9	18	7.8	16	7.0
High risk (children: ≥ 10, parents: ≥ 17)	5	2.2	26	11.3	16	7.0

Among mothers, the overall prevalence of suicidality (≥ 1 point) was 46.1% (*n* = 106), with 61 women in the lower risk group, 18 women with a moderate and 26 women with a high risk. The past-month prevalence of suicidal ideation was 37.4% (*n* = 86), while the rates for having a suicide plan and attempting suicide in the past month were 7.4% (*n* = 17) and 5.2% (*n* = 12). The lifetime prevalence of suicide attempt was 22.6% (*n* = 52).

The overall prevalence of suicidality (≥ 1 point) among fathers was 37.0% (*n* = 85). The majority (*n* = 53) of the men reporting suicidality belonged to the lower risk category, while 16 men each were in the moderate and high risk groups. The prevalence of suicidal ideation in the past month was 29.6% (*n* = 68). Of the eleven men (4.8%) who indicated having a suicide plan in the past month, four (1.7%) attempted suicide. In their lifetime, 27 men (11.7%) had ever attempted to take their lives.

### Factors associated with suicide risk

The results of the multinomial logistical regression analyses for children and parents are displayed in Tables 4 and 5, respectively. Among children, the variables significantly positively associated with both a lower and moderate/high suicide risk compared to no suicide risk were higher age as well as higher levels of externalizing problems and PTSD symptoms. Higher levels of internalizing problems were significantly related to a moderate or high risk relative to no risk. Neither the sociodemographic variables (e.g., gender, orphan status, household size and income) nor the environmental variables, i.e., exposure to war trauma, maltreatment by parents and social support, were significantly associated with children`s suicide risk. However, a follow-up analysis showed that when omitting children`s psychopathology, higher levels of maltreatment by parents and exposure to war trauma were significantly related to a lower and moderate/high suicide risk compared to no suicide risk, respectively.

**Table 4:** Multinomial logistic regression models predicting children`s current suicide risk

	Lower risk (*n* = 43)			Moderate/high risk (*n* = 14)		
	aOR	95% CI (LL, UL)	*p*	aOR	95% CI (LL, UL)	*p*
Age	**2.20**	**1.38, 3.51**	**0.001**	**3.03**	**1.15, 7.99**	**0.025**
Gender						
Male	1.02	0.47, 2.21	0.963	3.89	0.82, 18.51	0.088
Female (ref.)	1.00			1.00		
School attendance						
No	1.26	0.32, 4.92	0.739	0.80	0.05, 13.20	0.877
Yes (ref.)	1.00			1.00		
Orphan status						
Not orphan	1.46	0.46, 4.64	0.518	0.36	0.05, 2.39	0.288
Half or full orphan	1.00			1.00		
Household size	0.79	0.51, 1.22	0.285	1.05	0.50, 2.17	0.905
Household average income						
0–5000 TZS	0.67	0.21, 2.15	0.497	1.18	0.14, 7.99	0.968
5000–20,000 TZS	2.27	0.74, 7.02	0.154	1.70	0.21, 13.48	0.508
≥ 20,000 TZS	1.00			1.00		
Time spent in camp	1.08	0.61, 1.47	0.702	1.29	0.57, 2.91	0.541
Social support	0.95	0.60, 1.33	0.815	1.05	0.53, 2.08	0.896
Exposure to war-related trauma	0.88	0.56, 1.37	0.614	0.98	0.48, 1.97	0.949
Maltreatment by parents	1.31	0.90, 2.57	0.154	0.82	0.37, 1.80	0.615
Internalizing problems	1.29	0.85, 1.96	0.228	**2.88**	**1.33, 6.26**	**0.007**
Externalizing problems	**1.56**	**1.06, 2.31**	**0.026**	**3.03**	**1.42, 6.49**	**0.004**
PTSD symptoms	**1.64**	**1.05, 2.57**	**0.030**	**2.30**	**1.02, 5.16**	**0.044**

Significant associations are bold. The reference group is the no risk group (*n* = 171), *X*^2^ (28, *N* = 228) = 83.24, *p* < 0.001, Nagelkerke *R*^2^ = 0.41

*aOR*   adjusted odds ratio, *CI* confidence interval, *LL* lower limit, *UL* upper limit, *TZS* Tanzanian shillings, *PTSD*  posttraumatic stress disorder

**Table 5:** Multinomial logistic regression models predicting mothers’ and fathers’ current suicide risk

	Mothers^a^						Fathers^b^					
	Lower risk (*n* = 60)			Moderate/high risk (*n* = 44)			Lower risk (*n* = 52)			Moderate/high risk (*n* = 32)		
	aOR	95% CI (LL, UL)	*p*	aOR	95% CI (LL, UL)	*p*	aOR	95% CI (LL, UL)	*p*	aOR	95% CI (LL, UL)	*p*
Age	1.08	0.68, 1.71	0.752	0.67	0.38, 1.19	0.174	0.96	0.67, 1.39	0.838	0.83	0.51, 1.36	0.461
Years of formal education	1.08	0.71, 1.63	0.733	1.00	0.61, 1.65	0.996	0.74	0.41, 1.25	0.115	**0.58**	**0.34, 0.98**	**0.043**
Household size	**1.74**	**1.17, 2.57**	**0.006**	1.10	0.71, 1.73	0.666	1.13	0.80, 1.60	0.477	1.19	0.75, 1.90	0.464
Household average income p. month												
0–5000 TZS	**0.32**	**0.12, 0.84**	**0.021**	1.16	0.30, 4.46	0.829	0.45	0.18, 1.11	0.081	0.65	0.19, 2.32	0.510
5000–20,000 TZS	0.44	0.15, 1.27	0.130	1.25	0.29, 5.33	0.767	1.20	0.45, 3.20	0.714	2.24	0.58, 8.63	0.240
≥ 20,000 TZS (ref.)	1.00			1.00			1.00			1.00		
Time spent in camp	0.98	0.68, 1.38	0.893	1.02	0.69, 1.51	0.908	1.21	0.80, 1.82	0.365	0.88	0.54, 1.44	0.611
Instrumental social support	0.64	0.15, 2.75	0.544	**0.05**	** < 0.01, 0.58**	**0.017**	0.38	0.14, 1.03	0.058	**0.04**	** < 0.01, 0.44**	**0.008**
Trauma exposure	0.98	0.65, 1.48	0.932	1.08	0.69, 1.70	0.723	1.50	0.95, 2.36	0.081	**1.81**	**1.03, 3.19**	**0.041**
Current community violence	**1.97**	**1.30, 2.99**	**0.001**	**1.59**	**1.00, 2.52**	**0.049**	0.80	0.53, 1.22	0.309	1.21	0.73, 2.00	0.463
Psychological distress	1.37	0.90, 2.08	0.141	**1.67**	**1.05, 2.67**	**0.032**	1.26	0.77, 2.07	0.353	1.56	0.82, 2.91	0.177
PTSD symptoms	0.81	0.52, 1.26	0.355	1.26	0.77, 2.04	0.354	0.72	0.41, 1.25	0.238	0.86	0.44, 1.69	0.665
Substance use	1.26	0.92, 1.72	0.157	0.77	0.46, 1.29	0.323	1.01	0.70, 1.46	0.956	0.65	0.39, 1.07	0.088

Significant associations are bold, *aOR* adjusted odds ratio, *CI* confidence intervals, *LL* lower limit, *UL* upper limit, *TZS* Tanzanian shillings, *PTSD* posttraumatic stress disorder

^a^Reference group: no risk group (*n* = 123), *X*^2^ (24, *N* = 227) = 66.16, *p* < 0.001, Nagelkerke *R*^2^ = 0.29, ^b^Rreference group: No risk group (*n* = 142), *X*^2^ (24, *N* = 226) = 55.86, *p* < 0.001, Nagelkerke *R*^2^ = 0.2

Mothers living in larger households and experiencing more community violence had significantly higher odds of a lower suicide risk compared to no risk, whereas mothers with the lowest income were significantly less likely to have a lower suicide risk than mothers with the highest income. Mothers experiencing higher levels of current community violence and psychological distress were significantly more likely to have a moderate or high suicide risk, whereas mothers perceiving higher levels of instrumental social support had significantly lower odds of a moderate or high suicide risk. The sociodemographic variables age, years of formal education and time in camp were not significantly related to mothers` suicide risk. Similarly, neither exposure to war-related trauma nor PTSD symptoms contributed significantly to mothers’ suicide risk. However, the proportion of mothers meeting all diagnostic criteria of PTSD in the moderate/high risk group was more than twice as high (59%) than in the no risk (25%) and lower risk (29%) group. A similar pattern was observed among fathers (41% in the moderate/high, 30% in the lower and 26% in the no risk group).

None of the examined variables were significantly associated with the odds of having a lower suicide risk for fathers. Fathers perceiving more instrumental support from other community members and having more years of formal schooling were significantly less likely to belong to the moderate/high risk group. A higher exposure to war-related traumatic events was significantly related to a moderate/high suicide risk.

## Discussion

This study is one of the first to examine the prevalence and associated factors of suicidality among refugee children and parents living in refugee camps in a low-income setting. While this poses a challenge for putting the findings into the context of previous research, it also offers the opportunity to evaluate the applicability of findings from other settings and to spur future research with vulnerable and understudied populations in similar contexts.

### Prevalence of suicidality

Overall, we observed high prevalence rates of suicidal ideation among children and their parents, with one of ten children and one of three parents reporting passive or active forms of suicidal ideation in the past month. The high prevalence of passive forms of suicidal ideation especially among parents may reflect the feelings of hopelessness and loss of control about one’s life frequently reported by refugees living in resource-poor camps [43, 44]. For parents, the past-month rates of concrete suicidal thoughts (20.4% for mothers and 11.7% for fathers) are higher than the past-year rates found in community samples of adults in low- and middle-income countries [3, 45] and in the middle of the range of past-month rates (1.2–32.3%) reported by refugees in camps in previous studies [28]. For youth, the rate of 4.3% is comparable to the rates for current suicide ideation reported among non-refugee youth in Western high income countries, e.g., 3.6% in the United States [46] and 3.8% in Germany [47]. However, it is lower than the average 12-month prevalence rate of suicidal ideation of 20.4% reported for non-refugee samples of adolescents in African countries [13] and of 19.9–27% for Palestinian refugee youth living in camps in Jordan, Lebanon and Syria [48]. The comparison with other studies reporting a high variation of suicidal ideation (4.4–70%) for refugee youth living in camps is difficult because these studies did not clearly operationalize suicidal ideation and did not indicate timeframes [30].

Past-month prevalence rates of suicide plans and attempts were generally lower than the prevalence rates of specific suicidal thoughts. However, the high proportions of those participants with specific thoughts also reporting plans (more than one-third of mothers and fathers) and of those with a plan reporting suicide attempts (100% of children, about two-thirds of mothers and one-third of fathers) underscore the importance of timely identification and prevention. The observed prevalence of past-month suicide plans and attempts as well as lifetime suicide attempts among parents were higher than the 12-month rates found among community samples of adults in low- and middle-income countries [2, 45] and higher than the rates (timeframe 15 months to six weeks) of suicide attempts among displaced populations in camps [28]. While we did not identify any study reporting the prevalence of suicide plans and attempts among refugee children, the past-month and lifetime prevalence rates of suicide attempts among children in our sample are lower than those reported in other studies with non-refugee children in Sub-Saharan Africa [49–51]. The higher prevalence of the different forms of suicidality among women compared to men is in line with existing research that identified female gender as a risk factor for suicidal ideation and behavior among community samples in low- and middle-income countries [2, 3].

### Factors associated with children’s suicide risk

Children’s psychopathology, including PTSD symptoms, internalizing and externalizing problems, was significantly related to their current suicide risk, which is consistent with previous findings from non-refugee children in Western high-income countries [46, 52] and Sub-Saharan African countries [50, 53–55]. Key characteristics of internalizing and externalizing problems include low self-esteem and high impulsivity, respectively, which have been identified as risk factors for suicidality [5, 56]. Future research could aim to disentangle potential differential contributions of specific mental health problems within the broader range of internalizing (e.g., depression and anxiety symptoms) and externalizing psychopathology (e.g., conduct problems and attention problems). In line with previous research [4, 13], older youth had a significantly higher current suicide risk. This could be due to the multiple socioemotional, biological, and cognitive changes in adolescence that may increase adolescents` vulnerability to environmental stress. Girls were not more likely than boys to be at risk for suicide. It is possible that the gender differences observed among parents only manifest themselves at a later age when risk factors related to rigid gender norms and gender-based discrimination, including sexual abuse and domestic violence, unequal chore burden and exclusion from employment and education, are particularly salient [57].

In contrast to our hypothesis and previous evidence from non-refugee adolescents in Sub-Saharan Africa [50, 58, 59], we did not observe an association between the number of children’s close friends and their suicide risk. It may be that the perceived quality of friendships rather than only the quantity is a more adequate measure of social support for children in this setting. However, having close friends was not related to suicidal ideation among adolescents in Swaziland [60] and a study with adolescents in Ghana found that having more close friends was associated with higher odds of suicidal ideation and behaviour [61], pointing to the need for more research on how friendships may contribute to youth`s risk or resilience in relation to suicidality. Children’s current and past exposure to interpersonal violence, conceptualized as maltreatment by parents and war-related traumatic events, did not contribute to children’s suicide risk. This is inconsistent with a large body of research documenting consistent associations between experiencing interpersonal violence and an increased suicide risk in various contexts [4, 62–64]. However, a follow-up analysis suggests that the effects of child maltreatment and war exposure are mediated by children’s psychopathology.

### Factors associated with parents’ suicide risk

Mothers and fathers who perceived higher levels of instrumental social support were less likely to have a moderate or high current suicide risk, which is in line with evidence on the protective role of social support with regard to suicide [65, 66]. This suggests that being able to receive financial support from others in times of emergencies is a crucial resource within the camps. Interestingly, however, having a low income compared to a higher income was related to a reduced likelihood of exhibiting a lower suicide risk for mothers. While it is challenging to interpret this finding given the small numbers of participants in the suicide risk and higher income groups, it might imply that having better economic opportunities than the majority could also be problematic in a context where there is widespread poverty, for example, because it leads to envy and discrimination by community members. Mothers living in larger households were also more likely to have a lower suicide risk relative to no risk. As women are usually responsible for raising children and taking care of the household in Burundian society, this may be due to higher caregiving burden and/or higher material hardship. For fathers, having more years of formal education was associated with reduced odds of a moderate/high suicide risk, which is consistent with robust evidence establishing low education as a risk factor for suicidality [2, 3, 45]. In a patriarchal culture, higher formal education may be especially beneficial for men to navigate scarce opportunities for income-generating activities in the camps.

Higher exposure to current community violence was related to higher odds of a lower and moderate/high suicide risk for mothers, underscoring the need to create safe living conditions and prevent ongoing interpersonal violence within the camps. Among fathers, the independent link between exposure to past war-related trauma and suicidality even when considering psychopathology is consistent with previous studies with trauma-exposed individuals in different settings [67–69]. Future studies should examine the pathways through which past and current adverse experiences and stressors differentially contribute to women and men`s suicidality in refugee camps.

The finding that higher levels of psychological distress were significantly related to mothers’ current suicide risk aligns with previous findings suggesting a higher impairment due to mental health problems among female refugees [70–72]. In contrast to previous findings with refugee samples resettled in low- and high-income settings [73–75], PTSD symptoms were not related to mothers and fathers’ current suicide risk. Available evidence suggests that the observed suicide risk in people suffering from PTSD is in part due to co-morbid depression [76]. Thus, the fact that we considered psychological distress, which includes depressive symptoms, may account for the lack of an independent association between PTSD symptoms and the current suicide risk. Moreover, we used a dimensional measure of PTSD symptoms instead of the PTSD diagnosis. The relatively high percentage of parents meeting criteria for PTSD in the moderate and high risk groups suggests that clinical levels of PTSD symptoms are related to an increased current suicide risk. Parents` substance use did not contribute to their current suicide risk, which is at odds with previous evidence from low- and middle-income countries [2]. However, the low percentage of parents who reported using substances, presumably due to their limited availability in the camps, may explain this.

### Limitations

Several limitations should be noted. First, our findings are drawn from cross-sectional data which do not allow to establish causality between participants’ suicide risk and associated factors. Second, although our combined random and systematic sampling approach provided a sample that can be considered representative for the Burundian families in the three camps, the findings may not be generalizable to refugees living in other cultural and socioeconomic contexts. Third, the Burundian interviewers occasionally relied on ad-hoc translation of terms and concepts from Kiswahili to Kirundi if participants had difficulties to understand their exact meaning. Although the interviewers were trained to use the same expressions and terms, the lack of a standardized translation may have increased the risk of measurement bias. Fourth, the moderate or high suicide risk groups for children and parents were small and comprised individuals with different levels of risk, which needs to be considered when interpreting the findings. Fifth, the SDQ scales showed low internal consistency in our sample, suggesting difficulties of the instrument in differentiating between conceptually distinct types of children`s mental health problems. Sixth, it is possible that participants’ responses were influenced by biases related to recall and social desirability. However, interviewers aimed to reduce this by normalizing experiences and by emphasizing confidentiality. Seventh, we did not consider a range of psychological, e.g., personality traits, and social variables, e.g., intimate partner violence and peer conflicts, that may potentially contribute to children and parents’ suicide risk.

### Implications for research and practice

More epidemiological studies examining suicidality in refugee populations are needed. Longitudinal designs, albeit difficult to implement in volatile displacement situations, are needed to inform about trajectories of suicidal ideation and behavior, and to examine underlying mechanisms of suicidality. This will also help to elucidate differences between individuals who think about suicide without taking further action and those who continue to plan and attempt suicide. The present findings point to the complexity of the factors that may give rise to suicidality and future studies should include risk and protective factors on the individual, family, and community level. In particular, future studies should systematically examine the extent and modes of transmission of suicidality from parents to children in refugee families. Furthermore, more research is needed on potential barriers for reporting suicidal thoughts as well as on contextually relevant alternative help-seeking patterns and pathways.

In general, to make most efficient use of the scarce resources, suicide prevention programs for refugees in camps should be integrated in a wider system of mental health and psychosocial support that targets risk and protective factors on multiple socio-ecological levels [77]. It is paramount that members from the refugee community are involved in the design, implementation, and evaluation of activities to increase acceptance of the program and to reduce barriers for engagement. Building awareness and establishing locally appropriate referral pathways are key to identifying individuals at suicide risk. However, as our findings indicate that psychopathological symptoms are a strong risk factor for suicidality particularly among children and mothers, these activities should go hand in hand with screenings for mental health problems and suicidality, which can be rolled out at schools or physical health services in the refugee camps. Individuals at risk for suicide should be provided with appropriate effective interventions according to their needs. For instance, an intervention that combined contact and safety planning measures delivered by community volunteers to Sri Lankan refugees in a camp in Southern India showed initial effectiveness in reducing suicidal behaviours [78]. In addition to individual psychopathology, our findings suggest various social factors that multi-level programs should consider in preventing suicide among children and parents. Established risk factors for children`s psychopathology, in particular maltreatment by parents, should be reduced. Perceived instrumental support by community members was consistently negatively related to mothers and fathers’ suicide risk. Service providers should, therefore, aim to improve livelihoods and promote social support and cohesion among communities in the camps. Further, measures to reduce the occurrence of violence in the community should be prioritized. As our findings suggest a particular risk of suicidality among mothers, all activities should put a focus on the needs and vulnerabilities of women in the camps.

## Conclusions

The findings showed a high prevalence of suicidal ideation, planning and attempts among Burundian refugee parents, particularly mothers. The prevalence of suicidality among refugee children was lower than in previous studies with Sub-Saharan African youth. Older age and higher psychopathology were significantly related to a moderate or high current suicide risk among youth. High levels of perceived instrumental support by community members significantly reduced the likelihood of a moderate or high current suicide risk for both parents, while higher exposure to community violence and higher psychological distress was significantly associated with a moderate or high current suicide risk for mothers. More research is needed to understand the nature and course of suicidality among refugees living in camps. Suicide prevention programs in the camps should address current psychopathology as well as violence and social support within the community.

### Supplementary Information

Below is the link to the electronic supplementary material.

Supplementary file1 (DOCX 39 KB)

Supplementary file2 (DOCX 22 KB)

## Data Availability

The data supporting the findings are available from the authors upon reasonable request.
