# Supplementary material for: Prevalence of suicidality and associated factors of suicide risk in a representative community sample of families in three East African refugee camps
Source: Soc Psychiatry Psychiatr Epidemiol. 2023 Jun 5;59(2):245–59. doi: 10.1007/s00127-023-02506-z (PMC10838827; doi:10.1007/s00127-023-02506-z)
Supplement: Supplementary file 1 — Supplementary file1 (DOCX 39 KB) [file 127_2023_2506_MOESM1_ESM.docx]

Supplementary Table 1: Bivariate correlations between variables included in model predicting children`s current suicide risk

|  | *1* | *2* | *3* | *4* | *5* | *6* | *7* | *8* | *9* | *10* | *11* | *12* | *13* | *14* |
| --- | --- | --- | --- | --- | --- | --- | --- | --- | --- | --- | --- | --- | --- | --- |
|  |  |  |  |  |  |  |  |  |  |  |  |  |  |  |
| 1. Suicidality | - |  |  |  |  |  |  |  |  |  |  |  |  |  |
| 2. Age | .18** | - |  |  |  |  |  |  |  |  |  |  |  |  |
| 3. Gender^a^ | -.05 | .01 | - |  |  |  |  |  |  |  |  | . |  |  |
| 4. School attendance^b^ | -.05 | -.05 | -.11 | - |  |  |  |  |  | . |  |  |  |  |
| 5. Orphan status^c^ | .07 | .11 | .01 | -.16* | - |  |  |  |  |  |  |  |  |  |
| 6. Household size | .10 | .31*** | .08 | .18** | -.26 *** | - |  |  |  |  |  |  |  |  |
| 7. Household income^d^ | .10 | -.16* | .13* | - .04 | .16* | -.24 *** | - |  |  |  |  |  |  |  |
| 8. Length of stay in camp | .03 | -.02 | .01 | - .41*** | -.05 | .17** | .18** | - |  |  |  |  |  |  |
| 9. Social support | -.08 | .04 | .05 | .15* | -.07 | .15* | .11 | .06 | - |  |  |  |  |  |
| 10. War-related trauma | .03 | -.04 | -.01 | .06 | -.11 | .07 | .03 | -.18** | < .01 | - |  |  |  |  |
| 11. Parental maltreatment | .03 | .03 | -.09 | .03 | -.08 | < -.01 | -.16* | - .18** | -.02 | .15* | - |  |  |  |
| 12. PTSD symptoms | .34*** | .15* | -.11 | .05 | .11 | .07 | -.01 | .04 | -.05 | .15* | .24 *** | - |  |  |
| 13. Internalizing problems | .32*** | .07 | -.03 | .03 | -.02 | .03 | .04 | .14* | -.12 | .06 | .04 | .32*** | - |  |
| 14. Externalizing problems | .17* | -.03 | .09 | -.05 | .01 | .03 | .12 | .06 | -.01 | .05 | .13 | .20** | .34*** | - |

*Note:* All correlations involving child gender, school attendance, orphan status and household income are Spearmean correlations, all other correlations are Pearson correlations.

*** p ≤ .001, ** p ≤ .01, * p ≤ .05

^a^ 0 = male, 1 = female; ^b^ 0 = no, 1 = yes; ^c^ 0 = no, 1 = yes; ^d^ 0 = 0 – 5000 Tanzanian Shillings, 1 = 5000 to 20,000 Tanzanian Shillings, 2 = More than 20,000 Tanzanian Shillings

Supplementary Table 2: Bivariate correlations between variables included in model predicting mothers` current suicide risk

|  | *1* | *2* | *3* | *4* | *5* | *6* | *7* | *8* | *9* | *10* | *11* | *12* |
| --- | --- | --- | --- | --- | --- | --- | --- | --- | --- | --- | --- | --- |
|  |  |  |  |  |  |  |  |  |  |  |  |  |
| 1. Suicidality | - |  |  |  |  |  |  |  |  |  |  |  |
| 2. Age | -.08 | - |  |  |  |  |  |  |  |  |  |  |
| 3. Educational level | .05 | -.29*** | - |  |  |  |  |  | . |  |  |  |
| 4. Household size | .05 | .36*** | -.27*** | - |  |  |  |  |  |  |  |  |
| 5. Household income | .02 | -.14* | .18** | -.22*** | - |  |  |  |  |  |  |  |
| 6. Length of stay in camp | -.02 | .12 | .03 | .17** | .32*** | - |  |  |  |  |  |  |
| 7. Social support | -.21** | -.01 | .17* | .01 | .21*** | .18** | - |  |  |  |  |  |
| 8. War-related trauma | .17** | .03 | .16* | .07 | -.12 | -.04 | .03 | - |  |  |  |  |
| 9. Community violence | .31*** | -.11 | .13 | -.12 | < -.01 | -.08 | - .06 | .45*** | - |  |  |  |
| 10. PTSD symptoms | .33*** | .01 | .14* | .13* | -.09 | .04 | -.21** | .38*** | .34*** | - |  |  |
| 11. Psychological distress | .36*** | .15* | .01 | .12 | -.09 | .05 | -.06 | .28*** | .32*** | .59*** | - |  |
| 12, Substance use | -.04 | -.01 | -.06 | -.03 | -.16* | -.07 | -.07 | -.02 | .01 | -.01 | -.07 | - |

*Note:* All correlations involving household income are Spearmean correlations, all other correlations are Pearson correlations. *** p ≤ .001, ** p ≤ .01, * p ≤ .05

^a^ 0 = 0 – 5000 Tanzanian Shillings, 1 = 5000 to 20,000 Tanzanian Shillings, 2 = More than 20,000 Tanzanian Shillings

Supplementary Table 3: Bivariate correlations between variables included in model predicting fathers` current suicide risk

|  | *1* | *2* | *3* | *4* | *5* | *6* | *7* | *8* | *9* | *10* | *11* | *12* |
| --- | --- | --- | --- | --- | --- | --- | --- | --- | --- | --- | --- | --- |
|  |  |  |  |  |  |  |  |  |  |  |  |  |
| 1. Suicidality | - |  |  |  |  |  |  |  |  |  |  |  |
| 2. Age | -.10 | - |  |  |  |  |  |  |  |  |  |  |
| 3. Educational level | -.07 | -.29*** | - |  |  |  |  |  | . |  |  |  |
| 4. Household size | .02 | .21** | -.20** | - |  |  |  |  |  |  |  |  |
| 5. Household income^a^ | .10 | -.18** | .29*** | -.10 | - |  |  |  |  |  |  |  |
| 6. Length of stay in camp | -.07 | .13 | .03 | .05 | .13 | - |  |  |  |  |  |  |
| 7. Social support | -.18** | -.10 | .08 | -.07 | .15* | .12 | - |  |  |  |  |  |
| 8. War-related trauma | .24*** | .03 | .09 | .02 | .09 | -.08 | -.06 | - |  |  |  |  |
| 9. Community violence | .21** | -.14* | < .01 | -.01 | -.01 | -.30*** | - .17* | .37*** | - |  |  |  |
| 10. PTSD symptoms | .22*** | -.01 | .08 | .02 | -.04 | -.03 | -.05 | .45*** | .41*** | - |  |  |
| 11. Psychological distress | .29*** | -.03 | .05 | -.06 | -.02 | .08 | -.13* | .32*** | .26*** | .59*** | - |  |
| 12. Substance use | -.09 | .19** | -.16* | -.11 | -.07 | -.09 | -.08 | .16* | .19** | .06 | .02 | - |

*Note:* All correlations involving household income are Spearmean correlations, all other correlations are Pearson correlations. *** p ≤ .001, ** p ≤ .01, * p ≤ .05

^a^ 0 = 0 – 5000 Tanzanian Shillings, 1 = 5000 to 20,000 Tanzanian Shillings, 2 = More than 20,000 Tanzanian Shillings
