# Supplementary material for: Prevalence of suicidality and associated factors of suicide risk in a representative community sample of families in three East African refugee camps
Source: Soc Psychiatry Psychiatr Epidemiol. 2023 Jun 5;59(2):245–59. doi: 10.1007/s00127-023-02506-z (PMC10838827; doi:10.1007/s00127-023-02506-z)
Supplement: Supplementary file 2 — Supplementary file2 (DOCX 22 KB) [file 127_2023_2506_MOESM2_ESM.docx]

**Supplementary File 1:** Description of study measures

*Suicidality*

Parents’ and children’s suicidality was assessed using the Suicidal Scale of the Mini-International Neuropsychiatric Interview [MINI; 1] and the MINI for Children and Adolescents [MINI-KID; 2] respectively. The original scale consists of nine items which are answered with “No” or “Yes”. The items are weighted according to their estimated contribution to the suicide risk. Seven items refer to the past month and assess suicidal ideation (3 items; Yes = 1, 2 and 6 points), suicide plans (Yes = 8 points), active preparations for self-injury or a suicide attempt (Yes = 9 points), non-suicidal self-injury (Yes = 4 points) and suicide attempt (Yes = 10 points). One item assesses whether a person attempted suicide in their lifetime (Yes = 4 points). If at least one of the items about suicidal ideation is answered “Yes”, the frequency (occasionally, often, very often) and intensity (mild, moderate, severe) of the ideation are assessed and the respondent indicates whether they can control these thoughts or not (No = 8 points). If all items on suicidal ideation in the past month are answered “No”, the remaining questions referring to the past month are skipped, and the respondent is directly asked about suicide attempts in their lifetime. The points of all items are summed up to determine the respondent’s current suicide risk: low (1 to 8 points), moderate (9 to 16 points) or high ( ≥ 17 points). In the MINI-KID, the suicide scale consists of six items. Three items assess lifetime suicidal ideation (Yes = 1 point), self-injury (Yes = 2 points) and suicide attempt (Yes = 4 points). If all these items are answered “No”, the five following items referring to the past month are skipped. These items assess whether the child had a death wish (Yes = 1 point), wanted to hurt themselves (Yes = 2 points), thought about killing themselves (Yes = 6 points), suicide plans (Yes = 10 points) and attempted suicide (Yes = 10 points) in the past month. The current suicide risk is defined as low (1 to 5 points), moderate (6 to 9 points) or high ( ≥ 10 points).

*Sociodemographic characteristics*

We assessed parents` age, educational level, household size and average household income per month in Tanzanian Shillings. The latter variable was collapsed into the three categories: 0 to 5000 Shillings (0), 6000 to 20,000 Shillings (1) and more than 20,000 Shillings (2). Children answered purpose-built questions about their age, their current school attendance, and their orphan status (1 = No orphan/2 = Half- or full orphan).

*Social support*

Parents’ **instrumental** social support was assessed through two purpose-built questions: 1)*“If you suddenly needed a small amount of money – for example, enough to support your household for 1 week – how many people could you turn to who would be willing (not necessary capable) to provide this money?“* and 2) *“If you suddenly faced a long-term emergency, such as a harvest failure, how many people could you turn to who were willing (not necessary capable) to assist you?”*  The answer options for both questions were no one (0), one or two people (1), three or four people (2), five or more people (3) or an option for participants to indicate a specific number of people. The values for both questions were summed up. Children’s social support was operationalized through the purpose-built question “*How many good friends do you have?“*.

*Exposure to war-related traumatic events*

A checklist adapted from Ertl et al. [3] was used to assess children and parents’ lifetime exposure to various events they may have directly experienced, witnessed or perpetrated themselves in the context of war. The checklist for children contained 22 events and the checklist for parents consisted of 31 events. The checklist has been applied in previous studies with refugees in the Great Lakes region [4, 5]. Participants indicated whether they had experienced each event (1) or not (0). For the analysis all items were summed up to a total score of war exposure.

*Current experiences of violence*

Children’s current exposure to violence was operationalized as children’s self-reported experiences of maltreatment by their parents, which were assessed using the Parent-Child Conflict Tactic Scales [CTSPC; 6]. The 27 items cover specific acts of physical violence, emotional violence, non-violent discipline and neglect by mothers and fathers. Children indicate the frequency of each act in the past year on an 7-point Likert scale (0 = *never* *happened*; 1 = *once per year*; 2 = *twice per year*; 4 = *3 to 5 times per year*; 8 = *6 to 10 times per year*; 15 = *11 to 20 times per year*; 25 = *more than 20 times per year*). For the analysis, a sum score of child maltreatment by each parent was created by summing up all items except the four items on non-violent discipline and the sum scores for mother and fathers were combined to a total score of maltreatment (Cronbach’s alpha = 0.91). The CTSPC have been successfully used to assess children’s experiences of maltreatment by parents in East Africa [7, 8].

Parents’ reports of their directly experienced and witnessed incidents of community violence were used to operationalize parents’ current exposure to violence. The nine items were based on a study with Congolese refugees in Ugandan refugee camps and covered different types of violence, e.g. physical and sexual assault [5]. Parents indicated whether they had experienced or witnessed each event in the past month (1 = yes, 0 = No). The nine items were summed up for the analysis.

*PTSD symptoms*

Children’ PTSD symptoms according to the fifth edition of the Diagnostic and Statistical Manual of Mental Disorders (DSM-5) were assessed using the University of California at Los Angeles Child/Adolescent PTSD Reaction Index UCLA RI-5 [9]. Children reported on the frequency of PTSD symptoms in the past month over 31 items with a 5-point Likert scale ranging from 0 (*none of the time*) to 4 (*most of the time*). The UCLA RI-5 has demonstrated good psychometric properties in various cultural settings [10, 11] and it has been used in previous studies with refugee youth [12, 13]. The total score of PTSD symptoms created by summing up all items had a high internal consistency (Cronbach`s Alpha = 0.90).

The PTSD Checklist for DSM-5 [PCL-5; 14] was used to assess mothers and fathers` PTSD symptoms. Parents rated how much they were bothered by each of the 20 symptoms in the past month using a 5-point Likert scale ranging from 0 (not at all) to 4 (extremely). The responses to all items were summed up for the analyses. The PCL-5 has shown to be a psychometrically sound measure of PTSD symptoms [14] and has been successfully applied in studies with refugees [15, 16]. For the analyses, the sum score of all items was used, which had a high internal consistency both for mothers (Cronbach`s Alpha = 0.94) and fathers (Cronbach`s Alpha = 0.91).

*Other mental health problems*

Children`s internalizing and externalizing problems were assessed using the self-report version of the Strengths and Difficulties Questionnaire [SDQ; 17]. The SDQ consists of the five subscales emotional problems, peer problems, conduct problems, hyperactivity and prosocial behavior with five items each. The items are rated on a 3-point Likert scale from 0 (*Not at all true*) to 2 (*Certainly true*) regarding the past six months. The self-report version of the SDQ has demonstrated good psychometric properties [17] and it has been widely used in the context of Sub-Saharan African [18]. The subscales emotional problems and peer problems were combined into an internalizing problems score (Cronbach`s Alpha = 0.57) , while the subscales conduct problems and hyperactivity formed an externalizing problems score (Cronbach`s Alpha = 0.54). The poor internal consistency of the scores may be because the included subscales assess related, yet distinct constructs.

The 18-item version of the Brief Symptom Inventory [BSI-18; 19] served as a measure of parents` psychological distress. The BSI-18 assesses the three subscales depression, anxiety and somatization with six items each. Parents indicated how much they had been bothered by each symptom in the past seven days using a 5-point Likert scale from 0 (Not at all) to 4 (Extremely). The BSI-18 has shown good psychometric properties [20, 21] and has been used in previous studies with displaced populations [22, 23]. A sum score ranging from 0 to 68 was created by summing up all items except for the item assessing suicidal ideation. This score had high internal consistency both for mothers (Cronbach`s Alpha = 0.92) and fathers (Cronbach`s Alpha = 0.90).

In addition to psychological distress, we assessed parents’ substance use with the Alcohol, Smoking and Substance Involvement Screening Test (ASSIST) [24]. We considered the substance classes tobacco products, alcoholic beverages, cannabis and other substances the participants could specify (e.g. Khat). The ASSIST consists of eight items. The first item assesses lifetime use of each substance (0 = No, 3 = Yes), while the second item assesses use in the past three months on a scale from 0 (never) to 6 (Daily or almost daily). If respondents have used any substance in the past three months, they indicate using the same scale whether they had a strong desire to use the specific substance, whether the use led to any health, social, legal or financial problems and whether they failed to do what was expected from them in the past three months. Items six and seven assess whether a friend or relative has ever expressed concerns about the respondents` use of the substance and whether the respondents have ever tried to reduce or stop using the substance, respectively (0 = No, never, 6 = Yes, in the past three months, 3 = Yes, but not in the past three months). For the analysis we calculated the total substance involvement score by summing the responses to all items across all substances. This score had high internal consistency for mothers (Cronbach`s Alpha = 0.87) and fathers (Cronbach`s Alpha = 0.90).

*References*

1. Sheehan D V., Lecrubier Y, Sheehan KH, et al (1998) The Mini-International Neuropsychiatric Interview (M.I.N.I): The development and validation of a structured diagnostic psychiatric interview for DSM-IV and ICD-10. J Clin Psychiatry 59:22–33

2. Sheehan D V., Sheehan KH, Shytle RD, et al (2010) Reliability and validity of the mini international neuropsychiatric interview for children and adolescents (MINI-KID). J Clin Psychiatry 71:313–326. https://doi.org/10.4088/JCP.09m05305whi

3. Ertl V, Pfeiffer A, Saile R, et al (2010) Validation of a mental health assessment in an African conflict population. Psychol Assess 22:318–324. https://doi.org/10.1037/a0018810

4. Ainamani HE, Elbert T, Olema DK, Hecker T (2017) PTSD symptom severity relates to cognitive and psycho-social dysfunctioning - a study with Congolese refugees in Uganda. Eur J Psychotraumatol 8:. https://doi.org/10.1080/20008198.2017.1283086

5. Hecker T, Fetz S, Ainamani H, Elbert T (2015) The Cycle of Violence: Associations Between Exposure to Violence, Trauma-Related Symptoms and Aggression-Findings from Congolese Refugees in Uganda. J Trauma Stress 28:448–455. https://doi.org/10.1002/jts.22046

6. Straus MA, Hamby SL., Finkelhor D, et al (1998) Identification of child maltreatment with the Parent-Child Conflict Tactics Scales: Development and psychometric data for a national sample of American parents. Child Abuse Negl 22:249–70. https://doi.org/10.1016/S0145-2134(97)00174-9

7. Nkuba M, Hermenau K, Goessmann K, Hecker T (2018) Mental health problems and their association to violence and maltreatment in a nationally representative sample of Tanzanian secondary school students. Soc Psychiatry Psychiatr Epidemiol 53:699–707. https://doi.org/10.1007/s00127-018-1511-4

8. Ssenyonga J, Magoba Muwonge C, Hecker T (2019) Prevalence of family violence and mental health and their relation to peer victimization: A representative study of adolescent students in Southwestern Uganda. Child Abus Negl 98:104194. https://doi.org/10.1016/j.chiabu.2019.104194

9. Pynoos RS, Steinberg AM (2015) The University of California, Los Angeles, Post-traumatic Stress Disorder Reaction Index (UCLA PTSD Index) for the Diagnostic and Statistical Manual of Mental Disorders, 5th ed.; D. University of Californa, Los Angeles

10. Doric A, Stevanovic D, Stupar D, et al (2019) UCLA PTSD reaction index for DSM-5 (PTSD-RI-5): a psychometric study of adolescents sampled from communities in eleven countries. Eur J Psychotraumatol 10:1605282. https://doi.org/10.1080/20008198.2019.1605282

11. Takada S, Kameoka S, Okuyama M, et al (2018) Feasibility and psychometric properties of the UCLA PTSD reaction index for DSM-5 in japanese youth: A multi-site study. Asian J Psychiatr 33:93–98. https://doi.org/10.1016/J.AJP.2018.03.011

12. Karam EG, Fayyad JA, Farhat C, et al (2019) Role of childhood adversities and environmental sensitivity in the development of post-traumatic stress disorder in war-exposed Syrian refugee children and adolescents. Br J Psychiatry 214:354–360. https://doi.org/10.1192/bjp.2018.272

13. Grasser LR, Haddad L, Manji S, et al (2021) Trauma-Related Psychopathology in Iraqi Refugee Youth Resettled in the United States, and Comparison With an Ethnically Similar Refugee Sample: A Cross-Sectional Study. Front Psychol 12:704. https://doi.org/10.3389/FPSYG.2021.574368/BIBTEX

14. Blevins CA, Weathers FW, Davis MT, et al (2015) The Posttraumatic Stress Disorder Checklist for DSM-5 (PCL-5): Development and Initial Psychometric Evaluation. J Trauma Stress 28:489–498. https://doi.org/10.1002/jts.22059

15. Barbieri A, Visco-Comandini F, Fegatelli DA, et al (2019) Complex trauma, PTSD and complex PTSD in African refugees. Eur J Psychotraumatol 10:. https://doi.org/10.1080/20008198.2019.1700621

16. Ibrahim H, Ertl V, Catani C, et al (2018) The validity of Posttraumatic Stress Disorder Checklist for DSM-5 (PCL-5) as screening instrument with Kurdish and Arab displaced populations living in the Kurdistan region of Iraq. BMC Psychiatry 18:259. https://doi.org/10.1186/s12888-018-1839-z

17. Goodman R, Meltzer H, Bailey V (2003) International Review of Psychiatry The Strengths and Difficulties Questionnaire: a pilot study on the validity of the self-report version. Int Rev Psychiatry 15:173–177. https://doi.org/10.1080/0954026021000046137

18. Hoosen N, Davids EL, de Vries PJ, Shung-King M (2018) The Strengths and Difficulties Questionnaire (SDQ) in Africa: A scoping review of its application and validation. Child Adolesc Psychiatry Ment Health 12:1–39. https://doi.org/10.1186/s13034-017-0212-1

19. Derogatis LR (2000) BSI-18: Brief Symptom Inventory 18 - Administration, scoring, and procedures manual. Minneapolis, MN: NCS Pearson., Minneapolis, MN:

20. Franke GH, Jaeger S, Glaesmer H, et al (2017) Psychometric analysis of the brief symptom inventory 18 (BSI-18) in a representative German sample. BMC Med Res Methodol 17:. https://doi.org/10.1186/s12874-016-0283-3

21. Asner-Self KK, Schreiber JB, Marotta SA (2006) A cross-cultural analysis of the Brief Symptom Inventory-18. Cult Divers Ethn Minor Psychol 12:367–375. https://doi.org/10.1037/1099-9809.12.2.367

22. Rasmussen A, Nguyen L, Wilkinson J, et al (2010) Rates and Impact of Trauma and Current Stressors Among Darfuri Refugees in Eastern Chad. Am J Orthopsychiatry 80:227–236. https://doi.org/10.1111/j.1939-0025.2010.01026.x

23. Kaltenbach E, Härdtner E, Hermenau K, et al (2017) Efficient identification of mental health problems in refugees in Germany: the Refugee Health Screener. Eur J Psychotraumatol 8:1389205. https://doi.org/10.1080/20008198.2017.1389205

24. Humeniuk RE, Henry-Edwards S, Ali RL, et al (2010) The Alcohol, Smoking And Substance Involvement Screening Test (ASSIST). Manual for use in primary care. World Health Organization, Geneva
